# Disparities in access to gene therapy in the European Union: ethical and regulatory challenges

**DOI:** 10.1093/jlb/lsaf018

**Published:** 2025-09-30

**Authors:** Margaux Reckelbus, Riya Mohan, Phaedra Locquet, Eva Van Steijvoort, Isabelle Huys, Pascal Borry

**Affiliations:** Department of Public Health and Primary Care, KU Leuven, Leuven, Belgium; Department of Public Health and Primary Care, KU Leuven, Leuven, Belgium; Department of Public Health and Primary Care, KU Leuven, Leuven, Belgium; Department of Public Health and Primary Care, KU Leuven, Leuven, Belgium; Department of Public Health and Primary Care, KU Leuven, Leuven, Belgium; Department of Public Health and Primary Care, KU Leuven, Leuven, Belgium

**Keywords:** access, affordability, ethics, gene therapy, regulation, European Union

## Abstract

Gene therapies represent a significant advancement in modern medicine, offering potential cures for untreatable genetic disorders. However, equitable access to these innovative therapies remains a critical ethical challenge within the European Union (EU). This paper examines the adequacy of the EU’s centralized market authorization framework, supplemented by alternative pathways such as the Hospital Exemption and Compassionate Use Program, in addressing access disparities. While the centralized framework ensures high standards of safety, quality, and efficacy, its implementation reveals significant barriers related to affordability, geographical disparities, and fragmented national healthcare systems. High costs create financial obstacles for both healthcare systems and individuals, disproportionately affecting low-income countries and regions. Geographic disparities are further exacerbated by fragmented regulations and uneven healthcare infrastructures across member states, limiting patient access in rural areas. Alternative pathways, while designed to improve access, suffer from inconsistent national-level implementation. This paper argues that as the EU navigates the complexities of gene therapy regulation, it must focus on creating a more cohesive and inclusive framework. By doing so, it can ensure that the potential of gene therapies is realized in a manner that benefits all EU citizens, irrespective of their geographic or economic circumstances.

## I. INTRODUCTION

Over the past few years, there has been growing interest in gene therapies due to breakthroughs in genetic engineering technologies,^1^ such as CRISPR-Cas9 and similar editing systems.^2^ These advancements have made it possible to edit genes with greater precision, significantly increasing the potential for developing successful treatments.^3^ Increased investments from both public and private sectors in the EU have further fueled innovation, leading to a rise in clinical trials and new advancements in the field.^4^^,^^5^ As of 2023, over 300 clinical trials for gene therapies were ongoing in the European Union (EU), and since 2012, more than 15 gene therapies have received market authorization from the European Medicines Agency (EMA).^6^

The EMA has established a centralized market authorization framework for Advanced Therapy Medicinal Products (ATMPs), including gene therapies, to streamline the approval process across EU member states. This centralized procedure is rooted in a robust legal framework, based on Regulation (EC) No. 1394/2007 on ATMPs and Regulation (EC) No. 726/2004. These regulations ensure that ATMPs consistently meet high standards of quality, safety, and efficacy.^7^^,^^8^ That framework is supplemented with other instruments that foster market authorization for medicines toward unmet needs, such as conditional marketing authorizations,^9^ the accelerated assessment procedure, and the Priority Medicines (PRIME)^10^ scheme, all of which are designed to fast-track access to therapies for unmet medical needs.^11^

Additionally, exceptions to the market authorization framework like the Hospital Exemption (HE) pathway and the Compassionate Use Program (CUP) provide alternative access routes for patients in urgent need of gene therapies that have not yet received full market approval from the EMA.^12^^,^^13^ The HE pathway, regulated under Article 28(2) of the ATMPs Regulation No. 1394/2007, allows hospitals to administer ATMPs without formal market authorization under specific conditions.^14^ Similarly, the CUP, governed by Article 83 of Regulation (EC) No. 726/2004, offers an option for patients with life-threatening conditions who are unable to participate in clinical trials to access therapies prior to market approval.^15^ However, disparities in the national-level implementation of these exceptions can lead to unequal access for patients across EU member states.^16^^,^^17^^,^^18^ While these pathways address unmet medical needs, they also raise an important question: is the current market authorization framework, including these alternative routes, sufficient to ensure equitable access to gene therapies for all patients? The EU health policy, with its focus on protecting and improving health and ensuring equal access to modern and efficient healthcare for all Europeans, provides a relevant framework for considering this question.^19^

As gene therapies advance, the ethical challenge of fair access becomes increasingly urgent. Article 168 of the Treaty on the Functioning of the European Union (TFEU) obliges the EU to ensure a high level of health protection in all its policies.^20^ Similarly, Article 35 of the Charter of Fundamental Rights of the EU guarantees the right to preventive health care and medical treatment.^21^ These legal and normative commitments underscore the EU’s responsibility to address health inequities, particularly those related to access to innovative treatments. The principle of solidarity, central to the European social model, further reinforces the expectation of coordinated efforts to prevent unjust disparities.^22^ Yet, in the case of gene therapy, such solidarity appears to be challenged by structural barriers in the regulatory and reimbursement landscape.

Ensuring that patients have equitable access, regardless of geographic location or economic status, requires a thorough examination of existing EU regulatory frameworks and the alternative pathways available. It is essential to determine whether these frameworks effectively address patient accessibility.

This paper will explore whether the current EU regulatory framework for market authorization of gene therapies and its associated exceptions is ethically adequate in ensuring fair and equitable access for patients across the EU. We analyze this framework through the lens of access, treating it as a core ethical concern grounded in the principles of fairness, justice, and solidarity in the allocation of healthcare resources and the prioritization of patients.^23^ Gene therapies, while offering life-saving potential, often come with high costs, making access dependent on factors such as a patient’s country of residence, insurance coverage, or broader healthcare infrastructure. These disparities raise serious concerns about geographic and socioeconomic inequities^24^ and challenge the foundational ethical commitments of the EU, particularly the principle of equitable access to essential health services.^25^

This paper will investigate the ethical challenges surrounding patient access to gene therapies in Europe, evaluating whether existing EU regulatory frameworks for market authorization and the exceptions are sufficient to ensure that these innovative therapies reach all eligible individuals within the EU. Central to this analysis is the concept of patient access, viewed as a critical issue of social justice and fairness. Achieving equitable access means overcoming not only geographical and financial barriers but also ensuring that all patients, regardless of socio-economic status or location, have the chance to benefit from innovative treatments. This challenge is particularly pressing in the case of gene therapies because these therapeutics have life-saving potential, yet highly prohibitive prices.

The first part of our paper addresses high costs and affordability challenges, followed by an examination of geographic disparities across member states. The paper subsequently evaluates Health Technology Assessments (HTA) and cross-border healthcare initiatives designed to improve access. Furthermore, it examines alternative access pathways, such as HE and CUPs, to analyze their implications for improving equity in access. Taken together, these factors highlight the ethical complexities involved in ensuring equitable access to gene therapies.

## II. ECONOMIC AND GEOGRAPHICAL BARRIERS TO GENE THERAPY ACCESS

### II.A. High Costs and Affordability Challenges

After receiving formal market authorization, one of the primary barriers to accessing gene therapies in the EU is their high price. Treatments can range anywhere from €1 million to €2 million per patient. Products, like ®Luxturna and ®Zolgensma, exemplify this issue, as they are prohibitively expensive for our healthcare system, even in high-income European countries.^26^^,^^27^ In lower-income countries or those with more restricted healthcare budgets, such expenses are unsustainable and limit patient access even further. The high prices of gene therapies place a substantial financial burden on insurers, and governments, leading to ethical and distributive concerns about the sustainability of funding for such treatments and the implications for healthcare resource allocation overall.^28^^,^^29^

These high costs have exacerbated disparities in healthcare provision across EU regions. Although EU-level regulatory and funding initiatives exist to promote equitable access, countries with greater financial resources are better positioned to implement and sustain gene therapy programs, inadvertently widening the gap with lower-income Member States.^30^^,^^31^ This financial divide has also raised ethical questions about distributive justice: should substantial healthcare resources be directed toward costly gene therapies for a limited number of patients when these funds could address more prevalent health concerns?

In addition to the direct costs associated with gene therapy treatments, funding for these high-cost therapies can also divert resources away from other essential healthcare services. In all countries, constrained healthcare budgets force healthcare ministries to make challenging decisions about resource allocation. Governments must increasingly weigh the benefits of offering expensive, high-risk treatments for a few individuals against the needs of the broader population.^32^^,^^33^

One justification for the high prices of gene therapies has been attributed to the significant investments required for research and development (R&D).^34^ Gene therapies require years of development, challenging clinical trials, and rigorous post-market surveillance. Companies argue that the associated costs are reflected in the pricing of the final products. Patent protections and market exclusivity rights, particularly under the Orphan Drug Regulation (Regulation (EC) No. 141/2000), also play a major role in driving up prices. This regulation provides incentives to companies developing treatments for rare diseases, including 10 years of market exclusivity following drug approval. While this aims to encourage innovation in neglected disease areas, it can also foster monopolistic pricing that strains healthcare systems and inadvertently limits patient access.^35^^,^^36^^,^^37^

Further incentives offered by the Orphan Drug Regulation include reduced fees for applications, inspections, and post-authorization activities, as well as access to funding from both EU and national programs. While crucial for fostering development in areas where products risk not being profitable, such additional incentives also raise questions about the balance between promoting innovation and ensuring affordable access to life-saving treatments.^38^ Indeed, the current regulatory framework has sparked a strong debate about whether the incentives prioritize the financial interests of pharmaceutical companies over the public need for accessible treatments.^39^

While the high prices of gene therapies are often justified by their complexity and innovative nature, the actual net prices paid by countries are frequently reduced through negotiated discounts or managed entry agreements (MEAs). These confidential arrangements aim to balance affordability with the financial sustainability of healthcare systems. MEAs may include mechanisms such as rebates, pay-for-performance models, or risk-sharing agreements, tailored to a country’s specific budget constraints and healthcare priorities. For example, countries like Italy and Spain utilize hidden discounts or region-specific renegotiations to further lower the costs of ATMPs. While such strategies can help mitigate high upfront costs and improve national-level access, they also reflect a broader ethical issue—namely, price discrimination. This practice, where the same product is sold at different prices depending on the buyer’s characteristics or location, raises questions about fairness and justice in healthcare markets. As Elegido discusses, price discrimination can sometimes be ethically justifiable, especially when it enhances access for disadvantaged groups;^40^ however, in the EU context, it also risks reinforcing unequal access between Member States, especially when such negotiations lack transparency and coordination.^41^^,^^42^

Indeed, the lack of transparency in these pricing strategies raises ethical questions about procedural justice, specifically, whether patients and payers are able to fairly assess and influence the decisions that affect their access to care.

### II.B. Geographic Disparities and Fragmented Regulatory Frameworks

The EU’s fragmented regulatory framework exacerbates disparities in access to gene therapies. Each member state negotiates prices and determines reimbursement policies independently, leading to significant variability in patient access across the EU.^43^^,^^44^

High-income countries, like Germany, France, and the Netherlands, are often better equipped to subsidize gene therapies for their citizens. Their robust healthcare systems and innovative payment models facilitate access to these costly treatments, allowing patients to receive them more quickly and consistently.^45^ These innovative payment models spread the financial risk and tie reimbursement to treatment effectiveness over time, which is particularly useful given the high upfront cost and long-term uncertainties of gene therapies. This stands in contrast to traditional payment models, which typically involve a one-time lump-sum payment upon delivery of the treatment, regardless of patient outcome, placing a heavier burden on healthcare budgets.^46^ While high-income countries are generally better positioned to adopt and sustain these innovative approaches, they are nonetheless not immune to budget constraints and resource allocation pressures.^47^ For example, Germany’s healthcare system, despite its significant public funding, must still carefully allocate resources to ensure equitable access to all citizens. To settle on reimbursement programs, these countries use various resource allocation procedures. Germany employs outcome-based payment models, increasingly exploring approaches where payments for gene therapies are tied to their success, ensuring efficient allocation of healthcare funds. Additionally, Germany relies on HTAs, although pricing negotiations often take precedence. In France, HTAs play a central role in determining reimbursement levels within the national health insurance system, which covers a substantial portion of gene therapy costs. France also invests heavily in biomedical research, supporting specialized gene therapy centers that are equipped with necessary infrastructure and expertise. The Netherlands integrates HTAs into its reimbursement processes and employs innovative payment strategies as well, such as subscription-based and amortization models, to distribute costs over time, while fostering collaboration between public and private sectors to advance access to advanced therapies.^48^^,^^49^^,^^50^^,^^51^^,^^52^

In contrast, patients in lower-income countries, like Romania, Bulgaria, and Portugal, face greater challenges in accessing gene therapies. Romania, for instance, has one of the lowest healthcare expenditures per capita in the EU, leading to underfunded medical facilities and a shortage of healthcare professionals. While Romania’s healthcare system partially reimburses gene therapies through its positive drug list (PDL), not all therapies qualify for inclusion, further limiting patient access. Bulgaria similarly experiences chronic underfunding in its healthcare sector, with high out-of-pocket expenses for patients and challenges in maintaining healthcare infrastructure. Although Bulgaria also offers partial reimbursement for some therapies through a PDL, most gene therapies require individual access schemes, making them prohibitively expensive for most citizens.^53^ Portugal faces similar challenges, with a healthcare system constrained by chronic underfunding, resulting in staff shortages and long waiting lists. Patients in Portugal often face high out-of-pocket costs, further restricting access to gene therapies.^54^^,^^55^

Additionally, geographic disparities within member states can also lead to unequal access, particularly for patients in rural or remote areas. Even in countries with relatively advanced healthcare systems, rural populations still have less access to specialized care centers that can administer complex gene therapies. This urban–rural divide worsens the regional inequalities across the EU, leaving patients living in rural areas at a distinct disadvantage: first by their country’s overall healthcare system, and second by the distance they must travel to receive treatment. The logistical challenges of accessing gene therapies—especially given the intensive follow-up care and monitoring these treatments require—further restrict access for patients who are already marginalized by their geographic location.^56^^,^^57^^,^^58^

### II.C. Health Technology Assessments and Cross-Border Healthcare Initiatives

HTA bodies across the EU independently evaluate the cost-effectiveness of gene therapies. These assessments consider the value of treatments by weighing their costs against the benefits provided to patients and society at large.^59^ Although positive HTA recommendations can support reimbursement decisions, access to therapies remains inconsistent due to the fragmented nature of these reimbursement systems. As a result, financial constraints at the national level can lead to delays or prevent access to certain treatments, even with positive assessments.^60^

For instance, ®Zolgensma remains unavailable in some EU countries, including Poland and Romania, due to pricing and reimbursement challenges.^61^^,^^62^ Similarly, ®Luxturna is not available in Bulgaria and Romania, while ®Strimvelis is inaccessible in Bulgaria, Greece and Romania.^63^ This variation in access underscores the ethical implications of the current regulatory and reimbursement system, as patients in different countries are subjected to different levels of care and treatment availability. While it could be argued that healthcare is a national responsibility and that fairness only needs to be ensured within individual Member States, this perspective overlooks the ethical tensions that arise in a union committed to solidarity, equality, and cross-border cooperation. In a context where treatments are developed, approved, and often funded through EU-wide mechanisms, persistent disparities in access raise concerns about distributive justice and the equitable realization of patients’ rights across the Union.^64^

The EU’s Cross-Border Healthcare Directive (Directive 2011/24/EU) seeks to alleviate some of these disparities by allowing patients to seek treatment in other member states with EMA-approved therapies and to receive reimbursement from their home countries. In practice, however, this directive faces significant limitations. Patients often encounter complex reimbursement processes that vary by country, and out-of-pocket expenses, such as travel and accommodation costs, are prohibitive for many. Although the directive offers a potential solution for patients in lower-access countries, only a fraction of eligible patients can utilize it due to these financial and logistical hurdles. ^65^^,^^66^

### II.D. Alternative Access Pathways: HE and CUP

Alternative pathways like the HE and CUP offer potential access to gene therapies that lack formal marketing authorization. However, the absence of a unified regulatory framework complicates implementation. The HE pathway, which allows hospitals to administer ATMPs without formal approval is governed nationally. While it offers a route to treatment, the high manufacturing costs often exceed the capacities of already strained healthcare systems, placing the financial burden on patients. This creates inequities, limiting access to those who can afford these therapies. Moreover, HE demands substantial investment in infrastructure, expertise, and patient monitoring. Resources that are often scarce, particularly in rural or underfunded regions. These necessary investments often divert funds from other healthcare priorities, raising ethical questions about resource allocation.^67^^,^^68^^,^^69^^,^^70^ Similarly, the CUP, which grants access to investigational therapies before formal approval, varies widely across EU member states. Even when access is granted, many national healthcare systems are often unable to cover the associated costs for such treatments with uncertain outcomes, further exacerbating financial barriers to treatment.^71^^,^^72^^,^^73^^,^^74^

The HE pathway also exacerbates geographical disparities, as wealthier countries, with stronger healthcare infrastructures, are better equipped to implement it. These nations can finance clinical trials, patient monitoring, and manufacturing costs, allowing patients in these countries to benefit from access to unapproved but potentially life-saving therapies. In contrast, lower-income countries, often lacking the infrastructure and financial capacity, face significant hurdles in providing similar opportunities. This deepens the divide within the EU, where patients’ access to experimental treatments is largely dictated by national wealth, highlighting the tension between national healthcare sovereignty and the EU’s mission to ensure equal access to care (50, 51).^75^^,^^76^ Similarly, the CUP reflects previously discussed regional disparities because high-income nations are better positioned to support compassionate use due to more robust healthcare systems and funding. On the other hand, financial and infrastructural limitations in less affluent countries prevent equitable access, leaving patients in lower-income or rural areas without access to potentially lifesaving therapies. This inequality further widens the healthcare gap across Europe, underscoring the need for more coordinated efforts to bridge these divides.^77^^,^^78^^,^^79^

Pharmaceutical companies also play a significant role in shaping access to treatments through the CUP, primarily through their pricing decisions. While some companies view compassionate use as part of their corporate social responsibility, others may be deterred by financial and logistical challenges associated with providing therapies still under investigation. This dynamic introduces ethical risks: on one side, companies that offer treatments can be seen as philanthropic, on the other, patients eager for treatment may accept high costs and uncertain outcomes, accepting risks for unapproved therapies. These ethical concerns highlight the need for unified regulatory oversight and safeguards to ensure that compassionate use remains equitable and safe for all patients.^80^^,^^81^^,^^82^

## III. CONCLUSION

In conclusion, while the EU’s centralized market authorization framework for gene therapies provides a rigorous and harmonized process, the broader regulatory landscape remains fragmented. On the one hand, the centralized market authorization framework ensures that safety, efficacy, and long-term monitoring are consistently applied across all member states, offering patients reassurance that approved therapies meet stringent standards. This process represents a key merit of the EU’s approach, effectively balancing patient safety with the pressing need to make innovative treatments available to those who need them most.

On the other hand, several barriers undermine the equitable provision of gene therapies across EU member states. First, although alternative pathways, such as the HE pathway and the CUP, that aim to expedite access for patients with urgent medical needs, there are significant inconsistencies in their implementation across member states. While such decentralized implementation is intended to provide flexibility and fast access to potentially life-saving therapies, they seem to result in uneven treatment availability within a fragmented system. Patients living in countries with underdeveloped healthcare infrastructures may find themselves at a disadvantage, unable to access the therapies they require. This geographical disparity raises ethical concerns regarding fairness and equity in healthcare access, underscoring the need for a more coordinated approach.

Additionally, these geographical disparities also reflect deeper systemic issues within the EU, including fragmented regulations, uneven healthcare infrastructure, and varying national resources. Although initiatives like the Cross-Border Healthcare Directive aim to address these inequities, their effectiveness is hindered by financial and logistical constraints, leaving many patients—especially those in lower-income or rural areas—unable to benefit from the potential of gene therapies.

Furthermore, the affordability of gene therapies adds another complex layer to the already fragmented system. High costs, driven by monopoly pricing, extensive research and development expenses, and national-level reimbursement negotiations, vary widely across member states. Despite the EU’s efforts to ensure equitable healthcare access, the prohibitive cost of gene therapies limits patients access, particularly those in lower-income countries or regions. This financial barrier not only limits patient access to essential treatments but also challenges national healthcare systems that must balance limited budgets with the demand for cutting-edge medical therapies.

These challenges raise ethical concerns that touch upon the EU’s legal and normative commitments to health equity. Under Article 168 TFEU and Article 35 of the Charter of Fundamental Rights, the EU is obligated to ensure high standards of health protection and access to care. The persistent gaps in gene therapy access suggest that current frameworks may fall short of fulfilling these obligations. Furthermore, these disparities also challenge the principle of solidarity in EU healthcare systems. Solidarity requires not only symbolic commitment but also concrete actions that could enable all citizens, regardless of nationality or socioeconomic status, to benefit from treatments.

Overall, while the EMA’s centralized market authorization framework represents a positive step, the broader fragmentation in the legal framework that governs gene therapies requires further attention. One key question is how the EU can extend the strengths of its centralized approval process to other regulatory pathways, such as HE and CUP, without compromising the flexibility these pathways offer. This requires a balance between maintaining rigorous safety and efficacy standards and ensuring that patients receive timely access to potentially life-saving treatments.

Finally, developing mechanisms to harmonize access across member states is essential for creating a more equitable system. This could involve establishing clearer guidelines for HE and CUP pathways, ensuring that all patients, regardless of their location, have access to the same level of treatment and oversight. Addressing the affordability issue may also require collaborative efforts to negotiate fair pricing strategies and explore innovative funding solutions that can alleviate the financial burden on both patients and national healthcare systems.

## Footnotes

1 Nature Media & AnGes, *The Rise of Gene Therapies*, Nature, https://www.nature.com/articles/d42473-019-00141-4 (accessed Nov. 28, 2024).

2 CRISPR Therapeutics, *Gene Editing*, https://crisprtx.com/gene-editing (accessed Nov. 28, 2024).

3 McKinsey, *How Could Cell and Gene Therapies Change Healthcare?*  McKinsey, https://www.mckinsey.com/industries/life-sciences/our-insights/how-could-gene-therapy-change-healthcare-in-the-next-ten-years (accessed July 28, 2021).

4
Annalee Armstrong, *Big Pharma Partnerships, Record $22.7B Investment Raise Profile of Regenerative Medicine in 2021*, Fierce Biotech, https://www.fiercebiotech.com/biotech/big-pharma-partnerships-record-investment-raise-profile-regenerative-medicine-2021 (accessed Apr. 5, 2022).

5 Toby AuWerter, Jeff Smith & Lydia The, *Cell and Gene Therapy: Biopharma Portfolio Strategy*, McKinsey (2020), https://www.mckinsey.com/industries/life-sciences/our-insights/biopharma-portfolio-strategy-in-the-era-of-cell-and-gene-therapy.

6 Kestin N. Vokinger, Camille E. G. Glaus & Aaron S. Kesselheim, *Approval and Therapeutic Value of Gene Therapies in the US and Europe*, 30 Gene Ther. 756–60 (2023), https://doi.org/10.1038/s41434-023-00402-4.

7
*European Medicines Agency (EMA), Marketing-Authorisation Procedures for Advanced-Therapy Medicinal Products (2017)*, https://www.ema.europa.eu/en/human-regulatory-overview/marketing-authorisation/advanced-therapies-marketing-authorisation/marketing-authorisation-procedures-advanced-therapy-medicinal-products.

8 EuroGCT*, Standard Marketing Authorisation Pathway: Centralised Procedure for ATMPs*, https://www.eurogct.org/research-pathways/commercialisation/market-access-atmps/standard-marketing-authorisation-pathway (accessed Oct. 2, 2024).

9 European Medicines Agency (EMA)*, Conditional Marketing Authorization,*  https://www.ema.europa.eu/en/human-regulatory-overview/marketing-authorisation/conditional-marketing-authorisation (accessed Oct. 2, 2024).

10 European Medicines Agency (EMA)*, PRIME: Priority Medicines*, https://www.ema.europa.eu/en/human-regulatory-overview/research-development/prime-priority-medicines (accessed Oct. 2, 2024).

11 Shayesteh Fürst-Ladani et al., *Regulatory Aspects for Approval of Advanced Therapy Medicinal Products in the EU*, in 367 Drug Delivery and Targeting, 367–87 (M. Schäfer-Korting & U. S. Schubert eds., Springer Int’l Publ’g, 2024), https://doi.org/10.1007/164_2023_648.

12 European Medicines Agency (EMA), *Compassionate Use*, https://www.ema.europa.eu/en/human-regulatory-overview/research-development/compassionate-use (accessed Oct. 2, 2024).

13 Alliance for Regenerative Medicine*, Recommendations for the Use of Hospital Exemption*, http://alliancerm.org/wp-content/uploads/2020/10/ARM-position-on-HE-final-Oct-2020.pdf (2020).

14 EuropaBio*, EU ATMP Hospital Exemption: Position Paper*, https://www.europabio.org/wp-content/uploads/2021/01/2020_10_H_PP_Application-of-the-ATMP-Hospital-Exemption-in-the-EU.pdf (2020).

15 Andrew Cummins, *Compassionate Use Programs in Europe: All You Need to Know*, Sciensus, https://www.sciensus.com/en-us/knowledge-hub/compassionate-use-programs-in-europe-all-you-need-to-know/ (accessed June 7, 2024).

16 European Medicines Agency (EMA), *Questions and Answers on the Compassionate Use of Medicines in the European Union*, https://www.ema.europa.eu/en/documents/other/questions-and-answers-compassionate-use-medicines-european-union_en.pdf (accessed Oct. 2, 2024).

17 European Medicines Agency (EMA) & Comm. for Medicinal Prods. for Hum. Use (CHMP), *Guideline on Compassionate Use of Medicinal Products, Pursuant to Article 83 of Regulation (EC) No. 726/2004*, https://www.ema.europa.eu/en/documents/regulatory-procedural-guideline/guideline-compassionate-use-medicinal-products-pursuant-article-83-regulation-ec-no-7262004_en.pdf (2007).

18 Alliance for Regenerative Medicine, *supra* note 13.

19 European Union, *Public Health Support – EU Action*, https://european-union.europa.eu/priorities-and-actions/actions-topic/health_en (accessed Nov. 18, 2024).

20 Treaty on the Functioning of the European Union (TFEU), *Article 168*. https://eur-lex.europa.eu/legal-content/EN/TXT/?uri=CELEX:12012E168.

21 European Union, *Charter of Fundamental Rights of the European Union. Article 35: Health Care*. https://eur-lex.europa.eu/legal-content/EN/TXT/?uri=CELEX%3A12012P%2FTXT (2012).

22
Barbara Prainsack & Alena Buyx, Solidarity in Biomedicine and Beyond*.* Cambridge University Press (2017).

23 Bryanna Moore, *Ethically Permissible Inequity in Access to Experimental Therapies*, 14 Clin. Ethics. 1–8 (2019). https://doi.org/10.1177/1477750919839920

24 Lucia Perez-Kempner et al., *HTA44 Unlocking Equitable Access to Revolutionary Cell and Gene Therapies Across EU Member States*, 27 Value Health. S361−S362 (2024).

25 Norman Daniels, *Justice and Access to Health Care*, in The Stanford Encyclopedia of Philosophy (Edward N. Zalta ed., Winter 2017 Edition), https://plato.stanford.edu/archives/win2017/entries/justice-healthcareaccess.

26 David Paitraud, *Luxturna: Première Thérapie Génique dans la Dystrophie Rétinienne Héréditaire Associée au Gène RPE65*, Vidal, https://www.vidal.fr/actualites/23409-luxturna-premiere-therapie-genique-dans-la-dystrophie-retinienne-hereditaire-associee-au-gene-rpe65.html (2019).

27 Eric Sagonowsky, *Novartis Plots ‘Innovative’ Payment Models for Pricey Luxturna’s EU Launch*, *Fierce Pharma,*  https://www.fiercepharma.com/pharma/novartis-scores-eu-nod-for-gene-therapy-luxturna-plans-innovative-payment-models (2018).

28 Helen Collis, *Can Europe Afford the Next Generation of Medicines?*, Politico, https://www.politico.eu/article/europe-afford-next-generation-medicine/ (accessed Apr. 20, 2023).

29 Remap, *Pricing and Reimbursement of Cell and Gene Therapies in Europe: Outcomes and Novel Approaches*, Remap Consulting, https://remapconsulting.com/gene-therapy-atmps/access-outcomes-novel-approaches/ (accessed Sept. 21, 2022).

30 Noemia T. de Siqueira Filha et al., *The Economics of Healthcare Access: A Scoping Review on the Economic Impact of Healthcare Access for Vulnerable Urban Populations in Low- and Middle-Income Countries*, 21 Int’l J. Equity Health. 191 (2022), https://doi.org/10.1186/s12939-022-01804-3.

31 European Parliament, Directorate General for Parliamentary Research Services, *Addressing Health Inequalities in the European Union: Concepts, Action, State of Play: In Depth Analysis* (2020), https://data.europa.eu/doi/10.2861/567478.

32 European Parliament, *supra* note 31.

33 Joseph K. Ho et al., *Economic Evidence on Potentially Curative Gene Therapy Products: A Systematic Literature Review*, 39 Pharmaco Economics 995, 995–1019 (2021), https://doi.org/10.1007/s40273-021-01051-4.

34 Kevin Doxzen, *Gene Therapies Could Change Millions of Lives—So How Will We Pay for Them?*, *World Economic Forum*, https://www.weforum.org/agenda/2021/09/gene-therapies-rare-diseases-payment-affoardable-drugs-medicine/ (accessed Sept. 3, 2021).

35 Regulation (EC) n°141/2000 of the European Parliament and of the Council of 16 December 1999 on Orphan Medicinal Products, https://eur-lex.europa.eu/eli/reg/2000/141/oj/eng.

36 Steven G. Morgan et al., *Pricing of Pharmaceuticals is Becoming a Major Challenge for Health Systems*, 368 Bmj l4627 (2020), https://doi.org/10.1136/bmj.l4627.

37 Alberto Loche et al., *Opportunities and Challenges for Cell Gene Therapies in Pharmaceuticals and Medical Products in Europe*, McKinsey, https://www.mckinsey.com/industries/life-sciences/our-insights/a-call-to-action-opportunities-and-challenges-for-cgts-in-europe (2021).

38 Regulation 141/2000, *supra* note 35.

39 Annemieke Aartsma-Rus et al., *Orphan Medicine Incentives: How to Address the Unmet Needs of Rare Disease Patients by Optimizing the European Orphan Medicinal Product Landscape Guiding Principles and Policy Proposals by the European Expert Group for Orphan Drug Incentives (OD Expert Group)*, 12 Front. Pharmacol. 3, (2021), https://doi.org/10.3389/fphar.2021.744532.

40 Juan M. Elegido, *The Ethics of Price Discrimination*, 21 Business Ethics Quarterly 633–660 (2011).

41 Juan-Carlos Rejon-Parrilla et al., *Pricing and Reimbursement Mechanisms for Advanced Therapy Medicinal Products in 20 Countries*, 14 Front. Pharmacol. 2 (2023), https://doi.org/10.3389/fphar.2023.1199500.

42 Virginia Ronco et al., *Price and Reimbursement of Advanced Therapeutic Medicinal Products in Europe: Are Assessment and Appraisal Diverging from Expert Recommendations*, 14 J. Pharm. Pol’y Prac. 30 (2021), https://doi.org/10.1186/s40545-021-00311-0.

43 Steven G. Morgan et al., *supra* note 36.

44 Alberto Loche et al., *supra* note 37.

45 Emma Wager & Cynthia Cox, *Introduction—International Comparison of Health Systems, Kaiser Family Foundation*,https://www.kff.org/health-policy-101-international-comparison-of-health-systems/?entry=table-of-contents-introduction (accessed May 28, 2024).

46 Caroline Horrow & Aaron S. Kesselheim, *Confronting High Costs and Clinical Uncertainty: Innovative Payment Models for Gene Therapies*, 42 Health Aff. (Project Hope**)** 1532, 1532–40 (2023), https://doi.org/10.1377/hlthaff.2023.00527.

47 Hugo Ossandon et al., *Challenges for Gene Therapy in the Financial Sustainability of Health Systems: A Scoping Review*, 19 Orphanet J. Rare Dis. 243 (2024), https://doi.org/10.1186/s13023-024-03249-z.

48 Caroline Horrow & Aaron S. Kesselheim, *supra* note 46.

49 Jesper Jørgensen et al., *Outcomes-Based Reimbursement for Gene Therapies in Practice: The Experience of Recently Launched CAR-T Cell Therapies in Major European Countries*, 8 J. Market Access Health Pol’y 1715536 (2020), https://doi.org/10.1080/20016689.2020.1715536.

50 Jespere Jørgensen et al., *The Use of Innovative Payment Mechanisms for Gene Therapies in Europe and the USA*, 16 Regen. Med. 405, 405–22 (2021), https://doi.org/10.2217/rme-2020-0169.

51 Hugo Ossandon et al., *supra* note 47.

52 Michal Pochopień et al., *An Overview of Health Technology Assessments of Gene Therapies with the Focus on Cost-Effectiveness Models*, 9 J. Market Access Health Pol’y. 2002006 (2021), https://doi.org/10.1080/20016689.2021.2002006.

53 Kostadin Kostadinov et al., *Navigating Gene Therapy Access: The Case of Bulgaria in the Context of the EU Regulatory Landscape*, 12 Healthcare. 458 (2024), https://doi.org/10.3390/healthcare12040458.

54 Hospital Waiting Times Concerning Portuguese, The Portugal News, https://www.theportugalnews.com/news/2022-05-03/hospital-waiting-times-concerning-portuguese/66794 (accessed May 3, 2022).

55 Portugal Healthcare Staff Shortages and Strikes Will Constrain Healthcare Service Delivery Over the Medium Term, Fitch Sols, https://www.fitchsolutions.com/bmi/healthcare/portugal-healthcare-staff-shortages-and-strikes-will-constrain-healthcare-service-delivery-over-medium-term-29-07-2024 (accessed July 29, 2024).

56 European Parliament, *supra* note 31.

57 European Medicines Agency & the Committee for Medicinal Products for Human Use, *Guideline on Compassionate Use of Medicinal Products, Pursuant to Article 83 of Regulation (EC) n°726/2004*, https://www.ema.europa.eu/en/documents/regulatory-procedural-guideline/guideline-compassionate-use-medicinal-products-pursuant-article-83-regulation-ec-no-7262004_en.pdf (2007).

58 Claudio Jommi et al., *The Economic Impact of Compassionate Use of Medicines*, 21 BMC Health Servs. Rsch. 1303 (2021), https://doi.org/10.1186/s12913-021-07255-w.

59 European Medicines Agency, *Towards a Permanent Collaboration Framework for EMA and Health Technology Assessment Bodies* , https://www.ema.europa.eu/en/news/towards-permanent-collaboration-framework-ema-and-health-technology-assessment-bodies (2023).

60 Alberto Loche et al., *supra* note 37.

61 Kostadin Kostadinov et al., *supra* note 53.

62
*Important Information*, *Fundacja SMA*, https://www.fsma.pl/updates/important-information/ (accessed Oct. 29, 2024).

63 Kostadin Kostadinov et al., *supra* note 53.

64 Anniek de Ruijter et al., *Solidarity and Trust in European Union Health Governance: Three Ways Forward,* 46 Lancet Regional Health – Europe 101047 (2024), ISSN 2666–7762, https://doi.org/10.1016/j.lanepe.2024.101047.

65
*Directive 2011/24/EU of the European Parliament and of the Council of 9 March 2011 on the Application of Patients’ Rights in Cross-Border Healthcare*, 088 O.J. L (2011), http://data.europa.eu/eli/dir/2011/24/oj/eng.

66 European Commission, *Study on Enhancing Implementation of the Cross-Border Healthcare Directive 2011/24/EU to Ensure Patient Rights in the EU*, https://europeanlung.org/en/news-and-blog/cross-border-healthcare-in-the-eu-status-challenges-and-recommendations/ (2022).

67 Esteve Trias et al., *The Hospital Exemption Pathway for the Approval of Advanced Therapy Medicinal Products: An Underused Opportunity? The Case of the CAR-T ARI-0001*, 57 Bone Marrow Transplant 156, 156–59 (2022), https://doi.org/10.1038/s41409-021-01463-y.

68 EuropaBio, *Reform of the EU General Pharmaceutical Legislation Joint Position Paper on the Hospital Exemption Scheme for ATMPs*, https://www.europabio.org/reform-of-the-eu-general-pharmaceutical-legislation-joint-position-paper-on-the-hospital-exemption-scheme-for-atmps/ (2023).

69 Patrick T. Harrison & Theodore Friedmann, *Cost of Gene Therapy*, 30 Gene Ther. 737 (2023), https://doi.org/10.1038/s41434-023-00408-y.

70 Hugo Ossandon et al., *supra* note 47.

71 European Medicines Agency & the Committee for Medicinal Products for Human Use, *Guideline on Compassionate Use of Medicinal Products, Pursuant to Article 83 of Regulation (EC) n°726/2004*, https://www.ema.europa.eu/en/documents/regulatory-procedural-guideline/guideline-compassionate-use-medicinal-products-pursuant-article-83-regulation-ec-no-7262004_en.pdf (2007).

72 Gayathri Balasubramanian et al., *An Overview of Compassionate Use Programs in the European Union Member States*, 5 Intractable Rare Dis. Res. 244, 244–54 (2016), https://doi.org/10.5582/irdr.2016.01054.

73 Claudio Jommi et al., *The Economic Impact of Compassionate Use of Medicines*, 21 BMC Health Servs. Rsch. 1303 (2021), https://doi.org/10.1186/s12913-021-07255-w.

74 Erwin De Cock et al., *Status of Compassionate Use Programmes (CUP) in the EU-5 (France, Germany, Italy, Spain, United Kingdom)* (presented at ISPOR Europe 2019, Copenhagen, Denmark) (accessed Nov. 4, 2019).

75 Ander Izeta et al., *Regulation of Advanced Therapies in Europe: Are We on the Right Track?*, 30 Cell Stem Cell 1013, 1013–16 (2023), https://doi.org/10.1016/j.stem.2023.07.004.

76 Allison Hills et al., *An Assessment of the Hospital Exemption Landscape Across European Member States: Regulatory Frameworks, Use and Impact*, 22 Cytotherapy 772, 772.e1 (2020), https://doi.org/10.1016/j.jcyt.2020.08.011.

77 Gayathri Balasubramanian et al., *supra* note 72.

78 Claudio Jommi et al., *supra* note 73.

79 Erwin De Cock et al., *supra* note 74.

80 European Medicines Agency & the Committee for Medicinal Products for Human Use, *supra* note 58.

81 Dennis Akkaya, *Expanded Access Program (Compassionate Use) Frameworks in Europe*, myTomorrows, https://mytomorrows.com/blog/biopharma/expanded-access-program-compassionate-use-frameworks-europe/ (2021).

82
*PJ View: Inequity of Access to Medicines for Compassionate Use Must End*, 311 Pharm. J. 1, (Aug. 2023), https://pharmaceutical-journal.com/article/opinion/pj-view-inequity-of-access-to-medicines-for-compassionate-use-must-end.

